# Ginsenosides as Potential Natural Ligands of SLC3A2: Computational Insights in Cancer

**DOI:** 10.3390/life15060907

**Published:** 2025-06-04

**Authors:** Jing Lu

**Affiliations:** Division of General Education, Seokyeong University, Seoul 02173, Republic of Korea; lujing@skuniv.ac.kr

**Keywords:** ginsenosides, SLC family, SLC3A2, anti-cancer, molecular dynamics simulation

## Abstract

*Panax ginseng* has been used as a traditional Oriental medicinal herb. This research investigates the potential of ginsenosides, bioactive phyto compounds derived from *ginseng*, as ligands of the solute carrier (SLC) family, including SLC3A2, SLC7A6, SLC7A11, SLC7A5, SLC7A8, SLC43A1, LCN2, SLC7A9, SLC7A7, and SLC7A10 proteins—which are overexpressed in various cancers and linked to metastasis. Using molecular docking (MD), ginsenosides (Km, Ro, compound K (CK), Rk1, and Ra1) with high binding affinities to SLC3A2 were identified, exhibiting binding energies of −9.3, −9.1, −8.7, −8.0, and −7.7 kcal/mol, respectively. Further molecular dynamics simulations (MDSs) conducted using GROMACS revealed improved stability, flexibility, and dynamic behavior of the selected ginsenosides, predicting their potential as natural ligands to bind with SLC3A2. Though this computational prediction underscores these ginsenosides as promising candidates as natural ligands to bind and interact with SLC family proteins during anti-cancer therapies, further in vitro and in vivo studies are needed to validate these interactions and anti-cancer effects.

## 1. Introduction

Metabolic reprogramming is a hallmark of cancer, enabling tumor cells to adapt their metabolic pathways to sustain uncontrolled growth and survival. Amino acid transport systems are pivotal in this process, supplying essential nutrients for protein synthesis and activating key signaling pathways such as mTORC1 [1], which regulates protein translation and cell proliferation. Among these systems, membrane transporters have emerged as critical players in cancer progression, attracting significant attention in therapeutic research [2].

Among the key players in these processes is the solute carrier (SLC) family of membrane transporters, which regulate the transport of amino acids, ions, and other small molecules across cellular membranes. These transporters help maintain metabolic balance and support the rapid proliferation of cancer cells by meeting their increased nutrient demands [3,4].

A subset of SLC family members, including SLC3A2, SLC7A6, SLC7A11, SLC7A5, SLC7A8, SLC43A1, LCN2, SLC7A9, SLC7A7, BSG, and SLC7A10, are frequently overexpressed in multiple cancer types and are strongly associated with tumor progression, metastasis, and therapy resistance [5,6,7,8]. These transporters facilitate the uptake of critical nutrients such as glutamine, leucine, and cystine, enabling cancer cells to thrive in nutrient-limited tumor microenvironments. Their surface accessibility also makes them attractive targets for therapeutic intervention.

SLC3A2, also known as CD98hc, is of particular interest due to its central role as a heavy chain subunit that forms heterodimers with several light chain amino acid transporters (LATs), including SLC7A5 (LAT1) and SLC7A11 (xCT) [7,9]. This interaction facilitates the uptake of essential amino acids and plays a key role in mTOR signaling, redox homeostasis, and immune evasion—all of which contribute to cancer aggressiveness [10,11]. Notably, SLC3A2 has been found to be overexpressed in diverse malignancies, such as breast, prostate, glioblastoma, and hematological cancers, and is often correlated with poor prognosis and increased metastatic potential [6,12]. Beyond nutrient transport, SLC3A2 is involved in integrin signaling and cell adhesion, further enhancing its role in metastasis, tumor microenvironment remodeling [13], and promoting tumorigenesis [14].

Despite its pivotal role in tumor biology, SLC3A2 remains underexplored as a therapeutic target, particularly in the context of natural compound-based interventions. In recent years, natural products have gained considerable attention for their ability to modulate cancer-related targets with lower toxicity profiles and higher selectivity. Among these, ginsenosides, the primary active constituents of *Panax ginseng*, have emerged as promising candidates due to their broad spectrum of pharmacological properties, including anti-inflammatory, antioxidant, and anti-cancer effects [15,16,17].

Ginsenosides such as Km, Ro, compound K (CK), Rk1, and Ra (structures are shown in Table 1) have been shown to interfere with key molecular pathways in cancer cells, including cell cycle regulation, apoptosis induction, and suppression of angiogenesis and metastasis [18,19]. Recent computational and experimental studies have suggested that these compounds may also interact with transmembrane transporter proteins, including those of the SLC family, influencing nutrient uptake and redox homeostasis in cancer cells.

Direct research on ginsenosides inhibiting SLC3A2 is limited; however, both the ginsenoside regulation in mTOR signaling pathways [20,21] and the inhibition of SLC7A11 activity by physical binding of ginsenoside Rg5 [22] suggest a possible indirect effect on SLC3A2-mediated pathways. To provide a comparative framework for evaluating the binding behavior of ginsenosides, two reference ligands—dexamethasone and tyrosine—were included in this study. Dexamethasone, a natural glucocorticoid with a steroidal backbone structurally similar to ginsenosides, was selected to assess how ginsenosides perform relative to known steroid-based ligands. Tyrosine, on the other hand, was included based on multiple lines of relevance: it is a listed drug associated with the SLC3A2 gene in the GeneCards database (https://www.genecards.org/cgi-bin/carddisp.pl?gene=SLC3A2 (accessed on 28 August 2023)), and it is a known physiological substrate of the SLC3A2–LAT1 (SLC7A5) heterodimer, a transporter complex frequently overexpressed in cancer. This complex plays a key role in transporting large neutral amino acids such as tyrosine, thereby supporting tumor cell growth and metabolic reprogramming [10,23,24]. Including tyrosine as a reference ligand allowed us to evaluate whether ginsenosides can exhibit similar or improved binding behavior compared with native substrates or drug-associated compounds.

Given the central regulatory function of SLC3A2 and the increasing interest in plant-derived anti-cancer agents, exploring the binding potential of ginsenosides to SLC3A2 may provide new insights into natural ligand-based inhibition of amino acid transporters in cancer. This study aims to fill this gap by applying MD and MDS to evaluate the interaction between ginsenosides and SLC3A2, assessing their binding stability and dynamic behavior through post-MD analyses. These findings highlight the potential of ginsenosides as promising candidates for targeting SLC3A2 and disrupting cancer-associated metabolic pathways and could open novel avenues for anti-cancer drug discovery.

## 2. Materials and Methods

### 2.1. Targets of SLC Family and Ginsenoside Acquisition

The targets of the SLC family, including SLC3A2, SLC7A6, SLC7A11, SLC7A5, SLC7A8, SLC43A1, LCN2, SLC7A9, SLC7A7, and SLC7A10 along with BSG, were identified as potential target gene candidates. While BSG (Basigin) is not officially an SLC (solute carrier) family member by classification, it plays a critical and supportive role in the function of certain SLC transporters, especially those involved in amino acid transport; therefore, it is often studied alongside them. The GeneCards database (https://www.genecards.org/, accessed on 10 January 2025) provided comprehensive information on all annotated and predicted human genes related to the SLC family. The STRING database (https://string-db.org/, accessed on 10 January 2025) generated the protein–protein interaction (PPI) network.

### 2.2. Gene Ontology (GO) Analysis and Kyoto Encyclopaedia of Genes and Genomes (KEGG) Pathway Analysis

The GO analysis was utilized to determine target activities, which included molecular function (MF), biological function (BF), and cellular component (CC) analysis. The KEGG pathway analysis was used to clarify the possible signaling pathways connecting the final target against cancer. The enrichment plots are based on the *p*-value, which was defined as the gene ratio expressed differently to the total number of targets in a signaling pathway [25]. The EnrichR (https://maayanlab.cloud/Enrichr/, accessed on 12 January 2025) software [26] was used to conduct GO and KEGG enrichment analyses with a *p*-value < 0.05.

### 2.3. Protein–Protein Interaction Network Construction

The PPI network was created using the standard PPI setup, which includes a confidence score cutoff of 900, STRING with scientific evidence as the database, and *H. sapiens* as the organism. Then, the accuracy of the network was assessed, and it was concluded that the common nodes were the most likely hubs. Furthermore, using CytoNCA in Cytoscape version 3.7.2 [27], sub-PPI networks were built using the top 10% of degree centrality (DC) of the PPI network.

### 2.4. Expression Levels of the Target Proteins

The Human Protein Atlas database (HPA) (https://www.proteinatlas.org/, accessed on 15 January 2025) [28] aims to provide information on the expression and distribution of diverse human proteins in various tissues. Additionally, by integrating numerous omics technologies, the HPA database was created to map every human protein present in cells, tissues, and organs [29]. The expression levels of these hub targets in different cancers were investigated based on information from the HPA database.

### 2.5. Core Target Gene Expression and Survival Analysis

The GEPIA database (http://gepia.cancer-pku.cn/) (accessed on 20 January 2025) was employed to analyze expression of the top anti-ACC core target genes in adrenocortical cancer (ACC) using Gene Expression Profiling Interactive Analysis. Both overall survival (OS) and disease-free survival (DFS) analyses were conducted based on the expression of these ten core target genes. A *p*-value of less than 0.05 was considered statistically significant [30].

### 2.6. Ligand Selection and Preparation

In this study, a collection of 128 ginsenosides extracted from *P. ginseng* was meticulously chosen for analysis [31]. These compounds have garnered attention in prior research, notably for their anti-cancer properties, prompting an investigation into their potential against the SLC3A2 protein. To compile the necessary chemical structures, their structures were manually crafted utilizing ChemDraw professional 20.0., followed by their conversion into three-dimensional models. Subsequent steps involved transforming all selected compounds, alongside reference molecules, into Protein Data Bank (PDB) format with the help of Open Babel software 3.1.1. To facilitate molecular docking (MD) studies, these compounds were then processed into the pdbqt file format—a task achieved using the functionalities provided by AutoDock Vina 1.2.5 [32].

### 2.7. Selection and Preparation of Protein Targets

The selection and preparation of the protein targets for this study were methodically conducted through a comprehensive review of the existing literature, database inquiries, and an understanding of their significance in oncogenesis. The transporters SLC3A2 (PDB ID: 7DF1), BSG (PDB ID: 4U0Q), SLC7A5 (PDB ID: 7DSL), SLC7A6 (AF-Q92536-F1), LCN2 (PDB ID: 3DSZ), and SLC7A9 (PDB ID: 6YV1) were pinpointed as molecular targets based on their implicated role in cancer [3,5,10,33]. The three-dimensional (3D) conformations of each target protein in its unmodified state were acquired from the RCSB PDB database—except SLC7A6 (AF-Q92536-F1), which was obtained from AlphaFold—ensuring authenticity and relevance. The process of preparing these structures for analysis entailed several meticulous steps: the removal of all water molecules and bound inhibitors; the verification and rectification of any missing atoms; and the addition of hydrogen atoms and necessary charges to stabilize the protein. These modifications were executed using AutoDock Tools, culminating in each protein’s conversion to the pdbqt format and thus optimizing each one for subsequent docking studies [34,35]. The grid box dimensions and size parameters used for molecular docking are mentioned in Supplementary Table S1.

### 2.8. Molecular Docking

For the docking simulations in this research, Auto Dock Vina [32] was preferred over Auto Dock 4.2 due to its enhanced accuracy in predicting ligand–protein binding configurations. The study employed Auto Dock Vina to conduct a virtual screening of 128 selected ginsenosides (listed in the Supplementary File S1) and two benchmark drugs—dexamethasone and tyrosine—against the target protein SLC3A2. The compounds underwent ranking according to their docking scores, which reflect the binding energies between each ligand and the protein. Visualization of the ligand–receptor interactions was achieved using the academic versions of PyMOL and the BIOVIA Discovery Studio Visualizer. The high binding energy five ginsenosides were further used for other SLC transporter-related hub targets.

### 2.9. Molecular Dynamics Simulation (MDS) Studies

The top five high binding energy ginsenosides (Km, Ro, compound K (CK), Rk1, and Ra1) from the docking study were further analyzed through MDS over the span of 200 nanoseconds (ns) using GROMACS [36]. Swiss PARAM was utilized for generating the topologies of these ginsenosides. Each receptor–ligand complex was immersed in a periodic cubic box and solved with an explicit SPC water model; the charge neutrality of the system was maintained with Na^+^ or Cl^−^ ions. The systems underwent energy minimization and were equilibrated using both NVT and NPT ensembles, followed by a 200 ns molecular dynamics simulation (MDS). Analysis of the MD trajectories, including RMSD, RMSF, Rg, and hydrogen bond occurrences, was performed using GROMACS 2023.1 tools.

#### ADMET Studies

The assessment of ADMET properties is a pivotal step in early drug discovery, aimed at identifying any potential adverse effects of possible ligands. To this end, ADMETlab 2.0 [37] was used in this study to predict the absorption, distribution, metabolism, excretion, and toxicity (ADMET) profiles of the top five ginsenosides (Km, Ro, CK, Rk1, and Ra1). This evaluation helps in understanding the pharmacokinetic behavior and safety profile of these compounds early in the development process.

## 3. Results

### 3.1. Retrieval of Target Proteins from the SLC Family Linked with Cancer and from GO Analysis and KEGG Pathway Analysis

**Figure 1:**
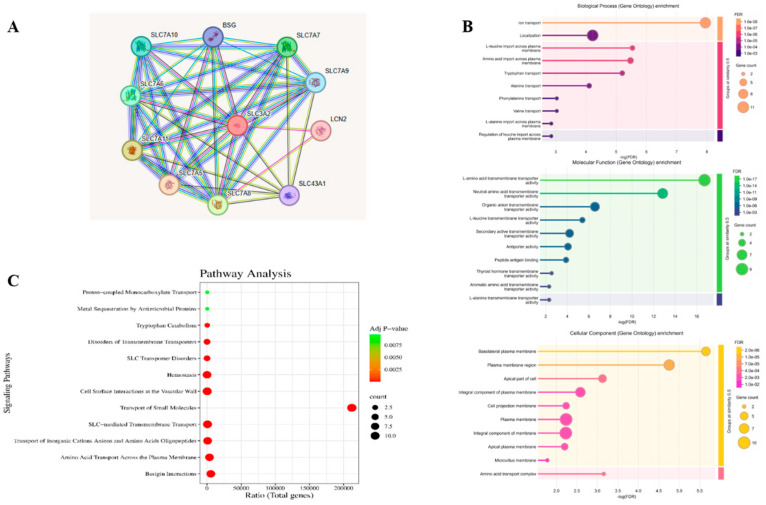
(**A**) The network analysis of SLC3A2 and its hub gene using the STRING database. (**B**) The biological functions (BFs), molecular functions (MFs), and cellular components (CCs) identified in the GO enrichment analysis of hub targets. (**C**) The KEGG pathways identified in the enrichment analysis of hub targets.

Eleven target proteins, including SLC3A2, SLC7A6, SLC7A11, SLC7A5, SLC7A8, SLC43A1, LCN2, SLC7A9, SLC7A7, and SLC7A10 along with BSG, were identified as potential targets that were closely related to the SLC family in the protein interaction network from the STRING database (Figure 1A). Therefore, these hub targets were used for GO analysis and KEGG pathway analysis. To further study the probable pathway and mechanism of the genes for cancer, GO and KEGG enrichment analyses were performed based on these hub targets. In the GO analysis, BP analysis depicted that the associated genes are initially centered on ion transport and localization. According to MF analysis, potential SLC family targets were mainly focused on L-amino acid, neutral amino acid, organic anion, and secondary transmembrane transporter activity. Cellular component (CC) analysis indicated that related targets were mainly centered on the plasma membrane, integral components of the membrane, the plasma membrane region, and the basolateral plasma membrane (Figure 1B).

Furthermore, the KEGG pathway analysis exhibited hemostasis, cell surface interactions at the vascular wall, transport of small molecules, SLC-mediated transmembrane transport, and BSG interactions. These signaling pathways were linked to cancer, and the expected targets were connected to these signaling pathways (Figure 1C).

### 3.2. Protein–Protein Interaction (PPI) Network and the Expression Levels of Hub Genes

**Figure 2:**
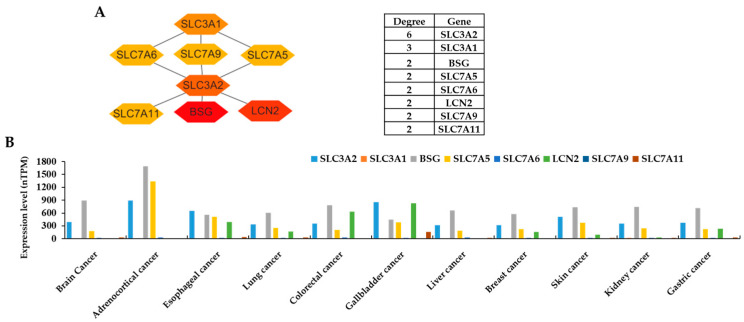
(**A**) PPI based on the degree value using Cytoscape. (**B**) RNA expression in different cancer cell lines were analyzed using the Human Protein Atlas database. The nTPM (number of transcripts per million) was used as the unit of expression levels. After analyzing the RNA expression, the genes with higher expression, including SLC3A2, BSG, SLC7A5, SLC7A6, LCN2, and SLC7A9, were selected.

In the PPI networks, the SLC family members interact with other targets consisting of 33 nodes and 35 edges. The top 10% degree centrality (DC) was created in the sub-PPI network of targets. Finally, eight related hub genes were obtained for further analysis (Figure 2A).

In order to analyze the functions of these targets in various cancers, first, the expression levels of the targets were detected in the HPA database. The results indicated that the SLC3A2, BSG, SLC7A5, SLC7A6, LCN2, and SLC7A9 genes showed higher expression in brain cancer, adrenocortical cancer, esophageal cancer, lung cancer, colorectal cancer, gallbladder cancer, liver cancer, breast cancer, skin cancer, kidney cancer, and gastric cancer, whereas the SLC3A1 and SLC7A11 genes showed lower expression in these cancer cases (Figure 2B). Therefore, six genes (SLC3A2, BSG, SLC7A5, SLC7A6, LCN2, and SLC7A9) were selected for further analysis and two genes (SLC3A1 and SLC7A11) were omitted.

### 3.3. Gene Expression and Survival Analysis of ACC Core Targets

**Figure 3:**
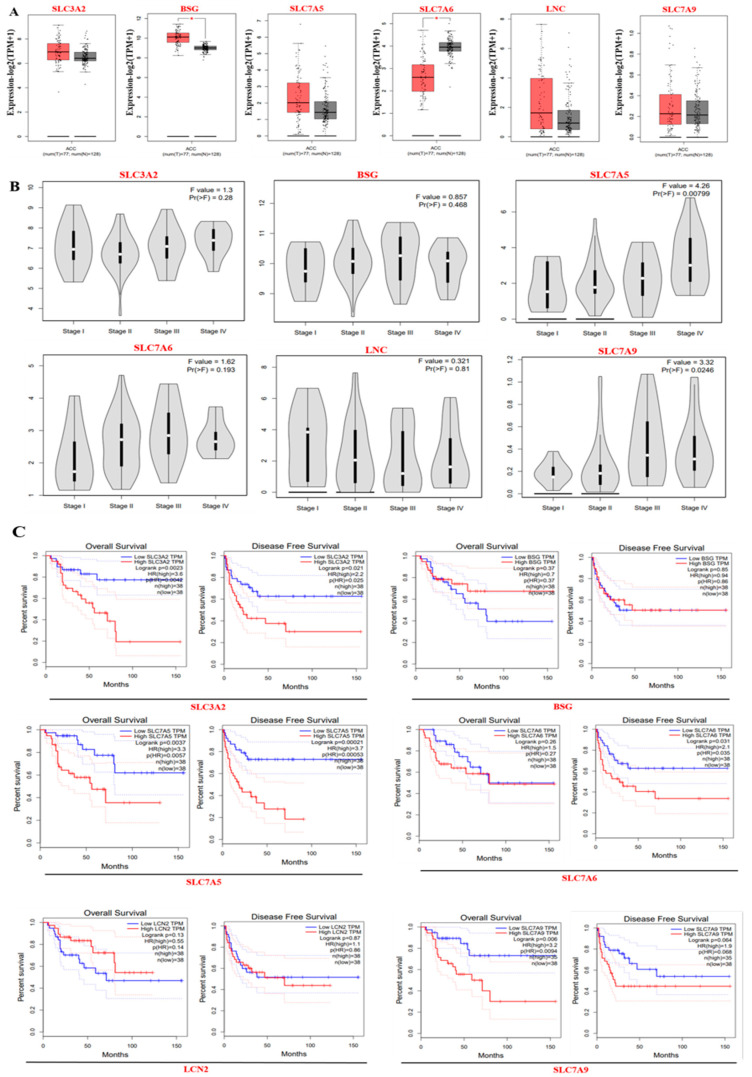
Expression of top core targets in adrenocortical cancer (ACC) and pathological stages; red- and gray-colored boxes represent tumor and normal cells, respectively. (**A**) Gene expression of the core target gene in ACC. (**B**) Core target gene expression level correlation with pathological stages of ACC based on TCGA data. (**C**) The correlation between core target gene expression and prognosis of patients with ACC tumors. There was a significant correlation between the upregulated gene expression of overall survival and disease-free survival of patients with tumors, as followed by a survival map. The * denotes that the difference in gene expression between two groups (usually tumor vs. normal tissues) is statistically significant. Specifically, this is based on a *p*-value threshold: * usually means *p* < 0.05. Sometimes additional asterisks are used: * → *p* < 0.05.

The expression levels of the top anti-ACC core targets SLC3A2, BSG, SLC7A5, SLC7A6, LCN2, and SLC7A9 in ACC and normal samples were analyzed using the GEPIA2 database. The differential expression of these targets in ACC versus normal samples indicates that they have various regulatory mechanisms in ACC (Figure 3A). Additionally, an analysis of cancer stages revealed a significant correlation between the expression levels of SLC3A2, BSG, SLC7A5, SLC7A6, LCN2, and SLC7A9 and the pathological stages of ACC, with *p*-values of 0.28, 0.468, 0.00799, 0.0237, 0.0359, 0.264, 0.193, 0.81, and 0.0246, respectively (Figure 3B). Moreover, the prognostic value of these core targets was also assessed using the GEPIA2 database. The upregulation of these six targets was significantly correlated with poor prognosis and OS in patients with ACC (Figure 3C). Similarly, the upregulation of these targets was significantly associated with poor prognosis and DFS in patients with ACC.

### 3.4. Molecular Docking-Based Virtual Screening

**Figure 4:**
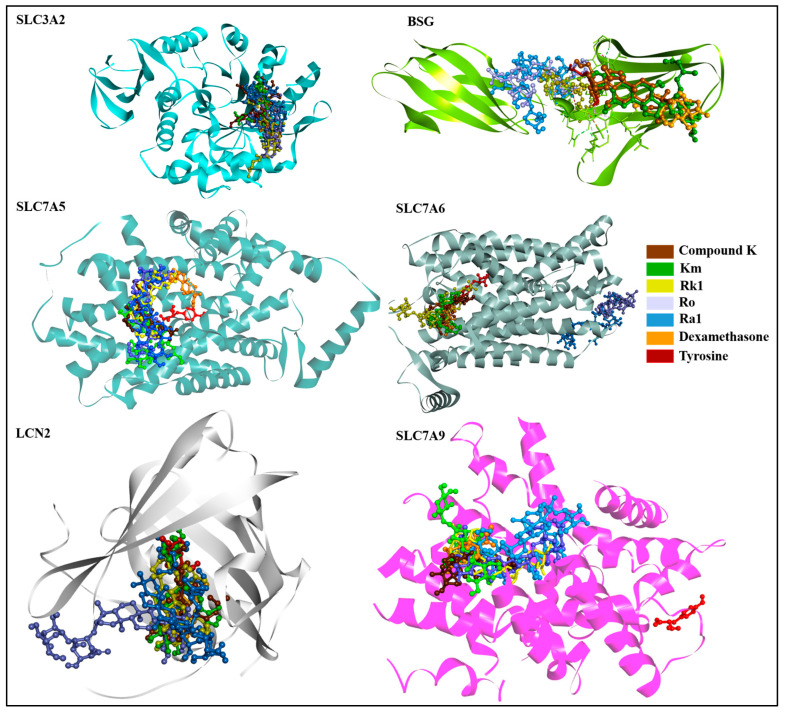
Interactions of ginsenosides and control drugs with SLC3A2, BSG, SLC7A5, SLC7A6, LCN2, and SLC7A9 presented in 3D format.

Utilizing MD as a pivotal technique in computer-aided drug discovery, first, the binding efficacy of 128 ginsenosides against SLC3A2 was evaluated, as this protein is highly and consecutively expressed in all cancers (Figure 2B). Then, the top five higher binding energy ginsenosides were used for docking with other SLC transporters, including SLC7A5, SLC7A6, LCN2, and SLC7A9 cancer-related targets, as well as BSG due to its involvement in amino acid transport. Dexamethasone and tyrosine served as controls. The findings demonstrated that ginsenoside Km depicted the highest energy with SLC3A2 (−9.3 kcal/mol). Moreover, ginsenoside Ro showed a strong affinity with BSG (−7.4 kcal/mol), SLC7A5 (−8.9 kcal/mol), and SLC7A6 (−8.2 kcal/mol) compared with the other ginsenosides and the control drugs. In the case of the LCN2 and SLC7A9 genes, ginsenosides Km and Rk1 showed the best negative energy at −8.6 kcal/mol and −9.3 kcal/mol, respectively. Table 2 and Figure 4 demonstrate the additional docking information and 3D interactions of targeted genes with ginsenosides, respectively. Supplementary Figures S1–S6 show the 2D interactions of ginsenosides with target proteins.

As shown in Table 2, all five ginsenosides exhibited strong binding energy with SLC3A2; therefore, further MDSs were performed using these ginsenosides with SLC3A2.

### 3.5. Dynamic Interactions and Stability Analysis of SLC3A2 Protein

**Figure 5:**
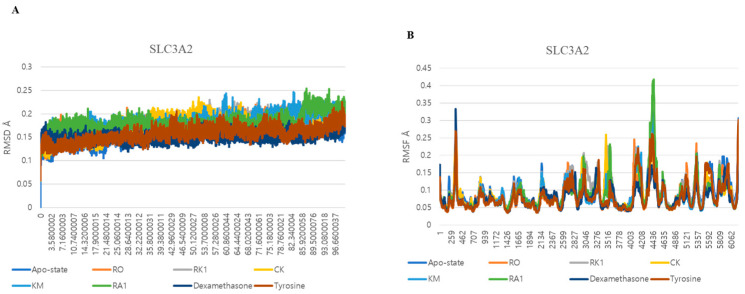
(**A**) RMSD of ginsenosides Ro, Rk1, CK, Km, and Ra1 and the control drugs (dexamethasone and tyrosine) in complex with SLC3A2. (**B**) RMSF of ginsenosides Ro, Rk1, CK, Km, and Ra1 and the control drugs (dexamethasone and tyrosine) in complex with SLC3A2.

The study extended into molecular dynamics simulations (MDSs), comparing the dynamic interactions of SLC3A2 with the top ginsenosides and control drugs over the span of 200 ns. Root mean square deviation (RMSD) analysis confirmed that all protein–ligand complexes reached equilibrium within the first 10–20 ns and remained stable throughout the simulation (Figure 5A). Among the ginsenosides, CK and Ra1 exhibited the lowest RMSD values (~0.15 nm), indicating tighter and more stable interactions with SLC3A2. Ro and Rk1 showed slightly higher but consistent RMSD values, remaining within an acceptable range. In contrast, the apo-state displayed greater structural deviation, emphasizing the stabilizing effect of ligand binding. The control drugs—dexamethasone and tyrosine—demonstrated RMSD patterns like CK and Ra1, supporting the reliability of the simulation setup.

**Figure 6:**
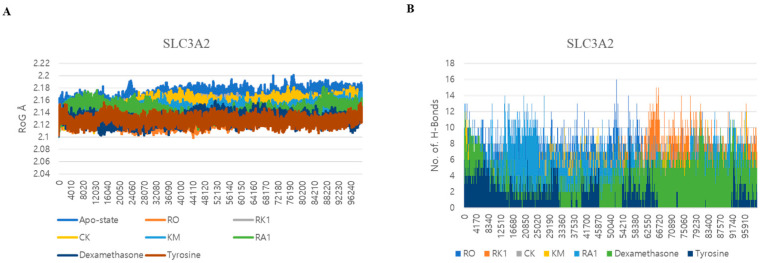
(**A**) Radius of gyration plots of MDS of ginsenosides Ro, Rk1, CK, Km, and Ra1 and the control drugs (dexamethasone and tyrosine) in complex with SLC3A2. (**B**) Line plots of ligand–protein H bonds for SLC3A2 with ginsenosides Ro, Rk1, CK, Km, and Ra1 and the control drugs (dexamethasone and tyrosine).

Root mean square fluctuation (RMSF) and radius of gyration (Rg) analyses provided further insights into the local flexibility and overall compactness of the complexes (Figure 5B and Figure 6A). RMSF analysis showed that CK, Km, and Ra1 complexes generally exhibited smoother and lower fluctuations than the apo-protein, indicating possible stabilization effects in flexible regions. These findings suggest that ligand binding contributes to local stabilization of SLC3A2. Radius of gyration analysis revealed that these same complexes maintained lower and more stable Rg values (~2.08–2.12 nm), indicating preservation of a compact and well-folded structure during the simulation. Conversely, the apo-state and the Ro complex both showed slightly higher Rg fluctuations, reflecting a less compact form.

Hydrogen bond analysis (Figure 6B) further supported the stability of the complexes. Ro and Km formed a higher number of hydrogen bonds throughout the simulation, while CK and Ra1 maintained moderate but stable interactions with the receptor. These observations are consistent with prior docking results, where these ligands exhibited strong binding affinities and key residue interactions. Overall, the MDSs validated the docking outcomes, confirming that the top-scoring ginsenosides—particularly CK, Km, Ro, and Ra1—establish stable and energetically favorable interactions with the SLC3A2 protein over time.

### 3.6. ADMET Study

**Figure 7:**
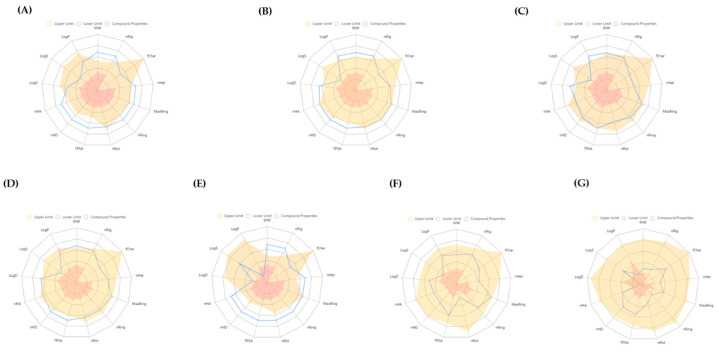
ADMET properties of (**A**) ginsenoside Ro, (**B**) ginsenoside Rk1, (**C**) ginsenoside CK, (**D**) ginsenoside Km, (**E**) ginsenoside Ra1, (**F**) dexamethasone, and (**G**) tyrosine. Abbreviations: MW—molecular weight; nRig—number of rigid bonds; fChar—formal charge; nHet—number of heteroatoms; MaxRing—number of atoms in the biggest ring; nRing—number of rings; nRot—number of rotatable bonds; TPSA—topological polar surface area; nHD—number of hydrogen bond donors; nHA—number of hydrogen bond acceptors; LogD—logP at physiological pH 7.4; LogS—log of the aqueous solubility; and LogP—log of the octanol/water partition coefficient.

In this comprehensive ADMET evaluation conducted using ADMETlab, the absorption, distribution, metabolism, and excretion (ADME) characteristics of the foremost five ginsenosides alongside the reference compounds dexamethasone and tyrosine were scrutinized (Figure 7). This assessment detailed critical physicochemical and biological attributes such as molecular weight, the count of rigid and rotatable bonds, formal charge, heteroatom quantity, total atom count, topological polar surface area, and hydrogen bond donors and acceptors, as well as logP, solubility, and partition coefficient at pH 7.4 (as illustrated in Figure 4 and Supplementary Tables S2–S7).

## 4. Discussion

The investigation into the anti-cancer potential of ginsenosides targeting SLC3A2 has yielded significant insights, aligning with and extending existing research in cancer therapeutics [38,39]. The MD and molecular dynamics simulations revealed a strong affinity and stability of ginsenoside–SLC3A2 complexes, suggesting a potent mechanism through which these natural compounds could exert anti-cancer effects. Notably, the binding energies observed for ginsenoside Ro [19,40] and its counterparts surpass those of traditional controls such as dexamethasone and tyrosine, reinforcing their potential as viable therapeutic candidates. Ginsenosides CK, Rk1, Km, and Ra1 [41,42,43] have been previously studied for their anti-cancer properties, demonstrating promising effects such as inducing apoptosis, inhibiting proliferation, and suppressing metastasis in various cancer models. These findings corroborate prior studies emphasizing the critical role of SLC3A2 in cancer cell proliferation, migration, and metastasis, further validating its suitability as a target for therapeutic intervention [33,44,45].

The structural similarities between the PPD (protopanaxadiol)-type ginsenosides and the PPT (protopanaxatriol)-type corticosteroids (such as dexamethasone) [46] are particularly noteworthy. These similarities underscore the potential of ginsenosides to mimic the pharmacological activity of synthetic steroids while potentially offering a safer profile with fewer side effects. This observation aligns with growing research into the molecular mimicry between plant-derived compounds and synthetic drugs, which has emerged as a promising avenue in drug discovery [47,48]. The steroid-like structure of ginsenosides also highlights their ability to modulate key signaling pathways, including integrin-dependent and mTORC1 signaling [21].

While in silico analysis presents a compelling case for the anti-cancer potential of ginsenosides [35,49], it also underscores the necessity for experimental validation. These early stages of in silico screening of possible ligands could accelerate the identification of promising candidates while minimizing late-stage failures.

The complexity of cancer biology and the dynamic nature of protein–ligand interactions require rigorous in vitro and in vivo studies to substantiate these computational predictions. Specifically, studies should focus on evaluating the inhibitory effects of ginsenosides on SLC3A2 activity in various cancer cell lines and animal models to confirm their efficacy and safety profiles.

Furthermore, exploring the synergistic effects of ginsenosides with existing chemotherapeutics could unveil new combination therapies that enhance therapeutic outcomes and better oral bioavailability while minimizing toxicity [50,51]. For instance, the ability of ginsenosides to modulate multiple signaling pathways makes them excellent candidates for combination regimens targeting both metabolic reprogramming and tumor microenvironment remodeling with existing chemotherapeutics and could unveil new combination therapies that enhance efficacy while minimizing toxicity. Beyond SLC3A2 inhibition, ginsenosides hold potential for broader applications in oncology. Their multitarget abilities, anti-inflammatory properties, and capacity to reduce oxidative stress position them as versatile agents in combating cancer and related conditions.

This study predicts the potential of ginsenosides in the context of cancer treatment, emphasizing the value of natural products in the quest for novel anti-cancer compounds. The converging paths of traditional medicine and modern drug discovery underscore the necessity for a multidisciplinary approach to harness the full spectrum of the benefits of ginsenosides. Future research should aim not only to validate these computational predictions but also to explore the broader pharmacological landscape of ginsenosides in cancer therapy.

## 5. Conclusions

SLC3A2 functions as a crucial mediator in the body by acting as a co-factor for light chain subunits like SLC1A5 and SLC7A11, facilitating their incorporation into the cell membrane. This process is pivotal for amino acid transportation, which is essential for tumor proliferation and managing oxidative stress. The computational research has predicted that the interaction ability of ginsenosides with SLC3A2 and other SLC transporters could modulate these transporters in combating various. The investigations through MD and molecular dynamics simulations reveal a robust interaction and the formation of stable complexes between ginsenosides and SLC3A2. The RMSD, RMSF, and Rg analyses all affirm the anti-cancer capabilities of ginsenosides, and the ADMET assessments predict their effective oral bioavailability. These insights underscore the therapeutic promise of ginsenosides as natural compounds for cancer treatment, highlighting the significant potential of leveraging traditional medicinal plants in the innovation of new oncological treatments. Experimental studies are recommended to confirm these in silico findings and fully explore the anti-cancer benefits of ginsenosides in relation to SLC3A2.

## Figures and Tables

**Table 1:** Structures of top five ginsenosides (Km, Ro, compound K (CK), Rk1, and Ra1).

KM	RO	Compound K (CK)	Rk1	Ra1
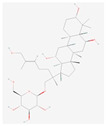	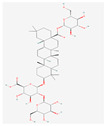	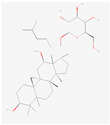	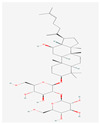	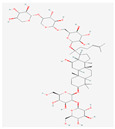
Leaf	Root, flower, fruit, leaf	Bioconversion of Root, fruit, leaf	Root (steamed) fruit, leaf	Root
C_36_H_62_O_10_	C_49_H_80_O_18_	C_36_H_62_O_8_	C_42_H_70_O_12_	C_58_H_98_O_26_

**Table 2:** Interaction of top five ginsenosides with amino acid residue of SLC family. Bold indicates the ginsenoside with highest binding energy for each proteins.

Targets	Compounds	Docking Score (kcal/mol)	Hydrogen Bonds	Other Bonds
SLC3A2 (7DF1)	Compound K (9852086)	−8.7	LYS75	ASP80 and TYR85
	**Km** (2754977172)	**−9.3**	GLU64, LYS71, and ASP77	LYS75
	Rk1 (11499198)	−8.0	GLU84, GLN100, and HIS102	0
	Ro (11815492)	−9.1	LYS57, TRP82, GLU84, and GLY103	0
	Ra1 (1009441542)	−7.7	SER78, ASP79, GLN81, SER193, and pro105	GLN100, GLY103, and VAL131
	Dexamethasone (5743)	−7.8	GLU64, LYS71, and LYS75	GLU73
	Tyrosine (6057)	−5.3	GLY161 and ARG247	ARG1258, PRO162, and ALA337
BSG (4U0Q)	Compound K (9852086)	−6.2	LYS75	ASAP80 and TYR85
	Km (2754977172)	−7.1	GLU64, LYS71, and ASP77	LYS75
	Rk1 (11499198)	−6.1	GLU84, GLN100, and HIS102	0
	**Ro** (11815492)	**−7.4**	LYS57, TRP82, GLU84, and GLY103	0
	Ra1 (1009441542)	−7	SER78, ASP79, GLN81, PRO105, and SER193	TRP82, GLN100, GLY103, and VAL131
	Dexamethasone (5743)	−4.8	GLU64, LYS71, and LYS75	GLU73
	Tyrosine (6057)	−6.5	LYS57 and ASP77	Val76 and ASP80
SLC7A5 (7DSL)	Compound K (9852086)	−7	THR73	0
	Km (2754977172)	−7.9	GLU309	PHE394
	Rk1 (11499198)	−8.6	GLY74	THR73, TYR248, LEu251, GLU309, ILE397, and TRP405
	**Ro** (11815492)	**−8.9**	GLU309 and ASN398	0
	Ra1 (1009441542)	−7.7	VAL70, GLU78, and THR154	THR73, GLU309, and PHE394
	Dexamethasone (5743)	−9.1	TYR248 and ASN398	SER401
	Tyrosine (6057)	−7	ILE63, GLY65, SER66, PHE252, and GLY255	SER342
SLC7A6 (AF-Q92536-F1)	Compound K (9852086)	−7.7	THR189 and ARG190	LYS42
	Km (2754977172)	−7.8	GLU44, LYS186, and ARG363	0
	Rk1 (11499198)	−7.5	LYS42 and LYS186	GLN40
	**Ro** (11815492)	**−8.2**	GLN153, PRO154, PRO157, ASP297, SER300, GLY314, SER317, and TRP318	0
	Ra1 (1009441542)	−7	ASN149, GLN153, ASP160, and ASP302	PRO157, ARG167, and G: U384
	Dexamethasone (5743)	−7.9	ARG341	SER340
	Tyrosine (6057)	−5.7	ASN49 and ASp193	ILE45
LCN2 (3DSZ)	Compound K (9852086)	−7	ARG36 and GLY38	ALA40, ALA41, and LYS125
	**Km** (2754977172)	**−8.6**	GLN54, VAL66, and PHE133	0
	Rk1 (11499198)	−6.9	GLY38, ALN39, MET81, SER134, and HIS165	ALA40 and PHE123
	Ro (11815492)	−8.2	ARG36, ALA41, LEU42, ASP47, PRO48, GLN49, LYS50, PHE71, LYS73, and HIS165	0
	Ra1 (1009441542)	−7.7	VAL66, LEU79, LYS125, SER127, and TYR132	0
	Dexamethasone (5743)	−7.1	GLY38	GLN54 and SER134
	Tyrosine (6057)	−5.4	VAL66, LEu79, and THR136	ALA68, MET81, TYR106, and PHE123
SLC7A9 (6YV1)	Compound K (9852086)	−8.5	SER116, ASN236, and GLN237	MET101 and PHE116
	Km (2754977172)	−8	THR91, ASN239, TYR358, and TYR389	0
	**Rk1** (11499198)	**−9.3**	ILE120, ASP233, and TYR386	ASN236 and GLN237
	Ro (11815492)	−8.3	GLY41, ILE44, TYR99, and ASP233	ASN236
	Ra1 (1009441542)	−7.3	ILE43, SER124, THR181, and GLN237	0
	Dexamethasone (5743)	−7.1	GLN237 and THR398	ASN236
	Tyrosine (6057)	−6.2	ALA138, TYR141, SER286, and ASP295	VAL142 and VAL291

## Data Availability

All of the data generated or analyzed during this study are included in the article (and its Supplementary Information Files).

## Supplementary Materials

The following supporting information can be downloaded at: https://www.mdpi.com/article/10.3390/life15060907/s1, Figure S1: 2D interaction of SLC3A2 with Compound K, Km, Rk1, Ro, Ra1. Dexamethasone and tyrosine were used as control drugs. Figure S2: 2D interaction of BSG with Compound K, Km, Rk1, Ro, Ra1. Dexamethasone and tyrosine were used as control drugs. Figure S3: 2D interaction of SLC7A5 with Compound K, Km, Rk1, Ro, Ra1. Dexamethasone and tyrosine were used as control drugs. Figure S4: 2D interaction of SLC7A6 with Compound K, Km, Rk1, Ro, Ra1. Dexamethasone and tyrosine were used as control drugs. Figure S5: 2D interaction of LCN2 with Compound K, Km, Rk1, Ro, Ra1. Dexamethasone and tyrosine were used as control drugs. Figure S6: 2D interaction of SLC7A9 with Compound K, Km, Rk1, Ro, Ra1. Dexamethasone and tyrosine were used as control drugs; Table S1: Grid box coordinates and size parameters used for molecular docking. Table S2: Parameters evaluated for drug-likeness of ginsenosides and the control drugs. Table S3: Parameters evaluated for absorption of ginsenosides and the control drugs. Table S4: Parameters evaluated for distribution of ginsenosides and the control drugs. Table S5: Parameters evaluated for metabolism of ginsenosides and the control drugs. Table S6: Parameters evaluated for excretion of ginsenosides and the control drugs. Table S7: Parameters evaluated for toxicity of ginsenosides and the control drugs; File S1: List of ginsenosides used and its binding score.
