# Supplementary material for: Ginsenosides as Potential Natural Ligands of SLC3A2: Computational Insights in Cancer
Source: Life (Basel). 2025 Jun 4;15(6):907. doi: 10.3390/life15060907 (PMC12194096; doi:10.3390/life15060907)
Supplement: Supplementary file 1 [file life-15-00907-s001.zip › Proof-Supplementary Tables.pdf]

Supplementary tables:

Table S1. Grid box coordinates and size parameters used for molecular docking.

Protein	Grid box center	Dimension Size	Exhaustiveness
SLC3A2 (2DH3)	x=21.318<br>y=62.807<br>z=47.065	x=59.972<br>y=67.662<br>z=57.296	8
BSG (4U0Q)	x=10.179<br>y=-26.157<br>z=-22.754	x=64.517<br>y=60.392<br>z=58.423	8
SLC7A5 (7DSL)	x=144.076<br>y=138.713<br>z=130.668	x=64.193<br>y=58.259<br>z=64.137	8
SLC7A6 (AF-Q92536_F1)	x=-14.908<br>y=-3.862<br>z= 4.791	x=101.671<br>y=73.235<br>z=87.770	8
LCN2 (3DSZ)	x=2.170<br>y=61.396<br>z=70.136	x=47.935<br>y=55.616<br>z=49.931	8
SLC7A9 (6YV1)	x=126.691<br>y=142.000<br>z= 113.358	x=45.294<br>y=53.568<br>z=62.533	8

Table S2. Parameters evaluated for drug-likeness of ginsenosides and the control drugs.

Compound	MCE-18	SAscore	Fsp^3	PAINS	NPScore	Pfizer
Ginsenoside Ro	176.69	6.29	0.91	0	2.39	Accepted
Ginsenoside Rk1	128.4	5.79	0.90	0	2.66	Accepted
Ginsenoside CK	110.4	5.40	0.94	0	2.83	Accepted
Ginsenoside Km	112.27	5.76	0.94	0	2.88	Accepted
Ginsenoside Ra1	197.05	7.26	0.96	0	2.00	Accepted
Dexamethasone	81.57	4.63	0.72	0	2.20	Accepted
Tyrosine	14.0	2.14	0.22	0	0.87	Accepted

Table S3. Parameters evaluated for absorption of ginsenosides and the control drugs.

Compound	Caco-2 permeability	MDCK permeability	Pgp-inhibitor	Pgp-substrate	HIA	F (20%)	F (30%)
Ginsenoside Ro	-6.13	0.0001	0.48	0.005	0.99	0.98	0.99
Ginsenoside Rk1	-5.34	8.2e-05	0.95	0.004	0.95	0.22	0.78
Ginsenoside CK	-4.94	3.1e-05	0.80	0.01	0.81	0.17	0.71
Ginsenoside Km	-5.06	6.3e-05	0.33	0.008	0.94	0.82	0.79
Ginsenoside Ra1	-6.07	0.0004	0.99	0.02	1.0	0.99	1.0
Dexamethasone	-4.82	2e-05	0.12	0.008	0.04	0.004	0.003
Tyrosine	-5.80	0.001	0.0	0.51	0.01	0.001	0.001

Table S4. Parameters evaluated for distribution of ginsenosides and the control drugs.

Compound	PPB (%)	VD (L/kg)	BBB (log BB)	Fu (%)
Ginsenoside Ro	81.19	0.20	0.09	11.91
Ginsenoside Rk1	91.23	0.71	0.08	8.01
Ginsenoside CK	93.57	1.06	0.05	5.59
Ginsenoside Km	83.73	0.77	0.09	10.68
Ginsenoside Ra1	50.38	-0.59	0.14	12.29
Dexamethasone	79.46	0.99	0.99	12.83
Tyrosine	25.19	0.42	0.15	77.46

Table S5. Parameters evaluated for metabolism of ginsenosides and the control drugs.

Compound	CYP1A2 inhibitor	CYP1A2 substrate	CYP2C19 inhibitor	CYP2C19 substrate	CYP2C9 inhibitor	CYP2C9 substrate	CYP2D6 inhibitor	CYP2D6 substrate	CYP3A4 inhibitor	CYP3A4 substrate
Ginsenoside Ro	0.0	0.054	0.0	0.09	0.0	0.1	0.0	0.095	0.012	0.004
Ginsenoside Rk1	0.0	0.075	0.001	0.29	0.0	0.097	0.0	0.126	0.013	0.055
Ginsenoside CK	0.001	0.13	0.002	0.845	0.009	0.229	0.0	0.141	0.032	0.099
Ginsenoside Km	0.001	0.105	0.001	0.375	0.002	0.086	0.0	0.084	0.035	0.06
Ginsenoside Ra1	0.0	0.02	0.0	0.047	0.0	0.034	0.0	0.052	0.001	0.001
Dexamethasone	0.012	0.608	0.025	0.802	0.024	0.129	0.01	0.072	0.425	0.914
Tyrosine	0.028	0.044	0.054	0.058	0.012	0.592	0.026	0.358	0.054	0.062

Table S6. Parameters evaluated for excretion of ginsenosides and the control drugs.

Compound	CL	$T_{1/2}$
Ginsenoside Ro	0.767	0.024
Ginsenoside Rk1	0.748	0.748
Ginsenoside CK	4.798	0.029
Ginsenoside Km	1.416	0.056
Ginsenoside Ra1	-0.18	0.022
Dexamethasone	3.363	0.765
Tyrosine	18.458	0.829

Table S7. Parameters evaluated for toxicity of ginsenosides and the control drugs.

Compound	hERG Blockers	H-HT	DILI	AMES Toxicity	ROA	Skin Sensitization	Carcino genecity	Eye Corrosion	Respiratory Toxicity
Ginsenoside Ro	0.001	0.18	0.022	0.089	0.317	0.001	0.018	0.003	0.037
Ginsenoside Rk1	0.014	0.236	0.007	0.066	0.066	0.011	0.008	0.003	0.70
Ginsenoside CK	0.046	0.27	0.006	0.041	0.173	0.064	0.007	0.003	0.929
Ginsenoside Km	0.036	0.207	0.009	0.041	0.306	0.039	0.005	0.003	0.776
Ginsenoside Ra1	0.018	0.117	0.003	0.059	0.131	0.001	0.006	0.003	0.017
Dexamethasone	0.033	0.543	0.018	0.04	0.398	0.041	0.774	0.003	0.95
Tyrosine	0.089	0.165	0.013	0.023	0.728	0.294	0.141	0.009	0.156
