# Supplementary material for: Ginsenosides as Potential Natural Ligands of SLC3A2: Computational Insights in Cancer
Source: Life (Basel). 2025 Jun 4;15(6):907. doi: 10.3390/life15060907 (PMC12194096; doi:10.3390/life15060907)
Supplement: Supplementary file 1 [file life-15-00907-s001.zip › Supplementary figure-Revised with 1200dpi.pdf]

SLC3A2

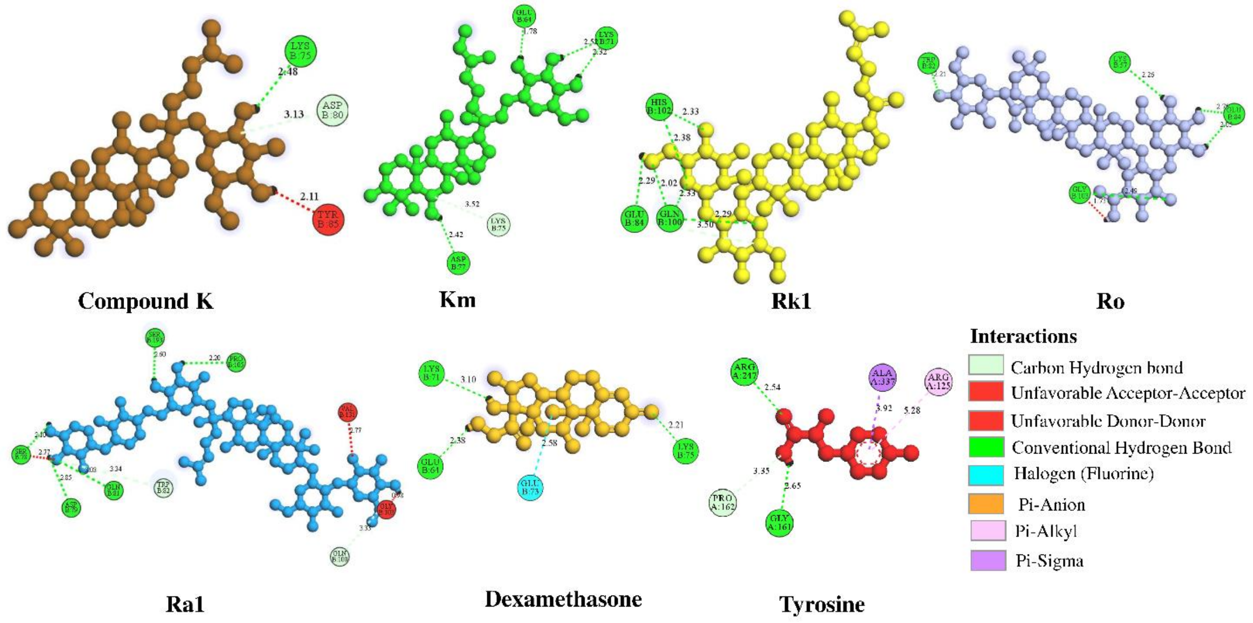

Figure S1. 2D interaction of SLC3A2 with Compound K, Km, Rk1, Ro, Ra1. Dexamethasone and tyrosine were used as control drugs.

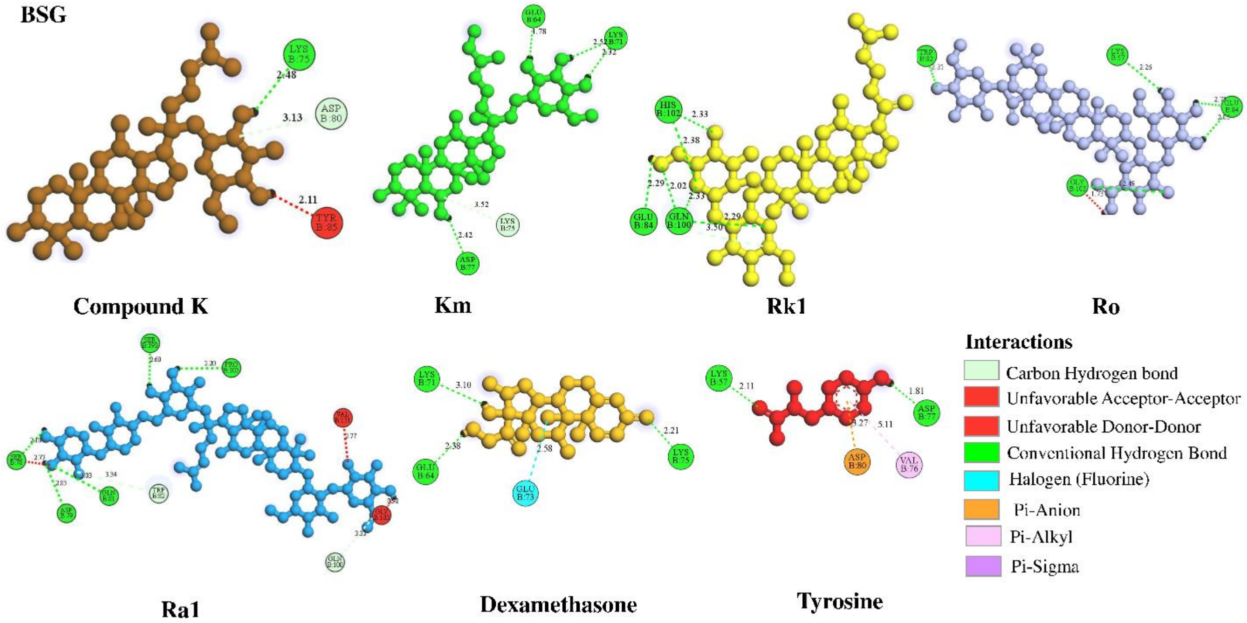

Figure S2. 2D interaction of BSG with Compound K, Km, Rk1, Ro, Ra1. Dexamethasone and tyrosine were used as control drugs.

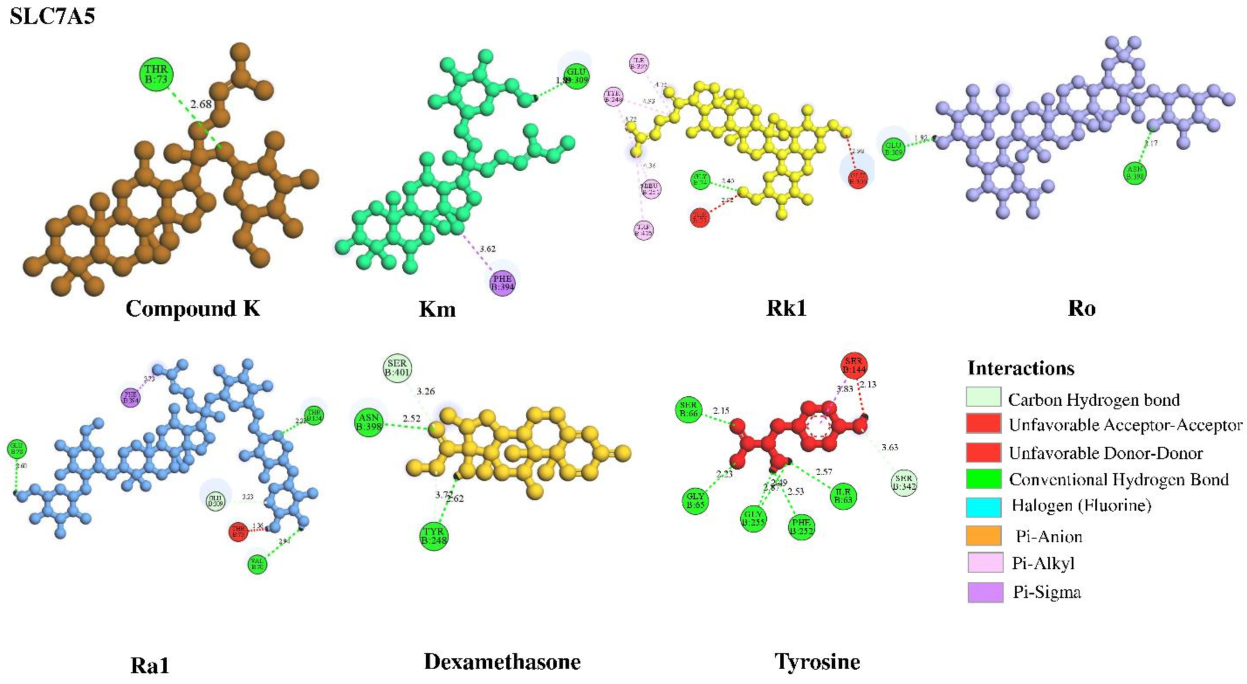

Figure S3. 2D interaction of SLC7A5 with Compound K, Km, Rk1, Ro, Ra1. Dexamethasone and tyrosine were used as control drugs.

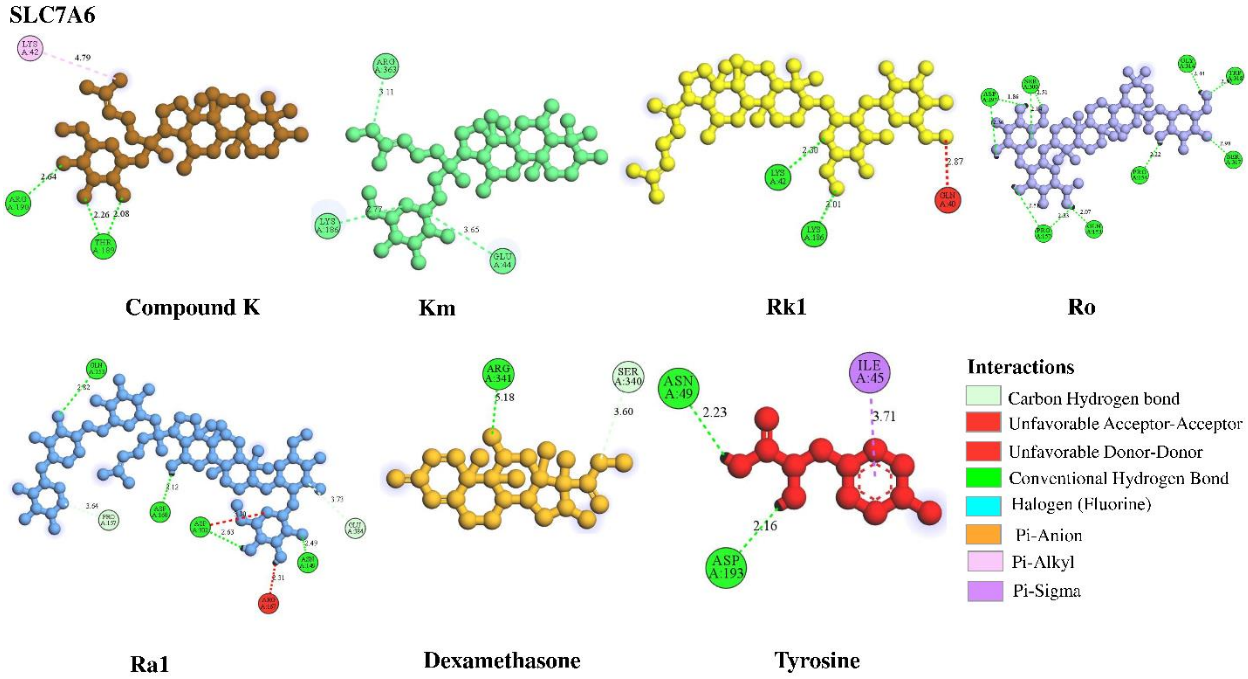

Figure S4. 2D interaction of SLC7A6 with Compound K, Km, Rk1, Ro, Ra1. Dexamethasone and tyrosine were used as control drugs.

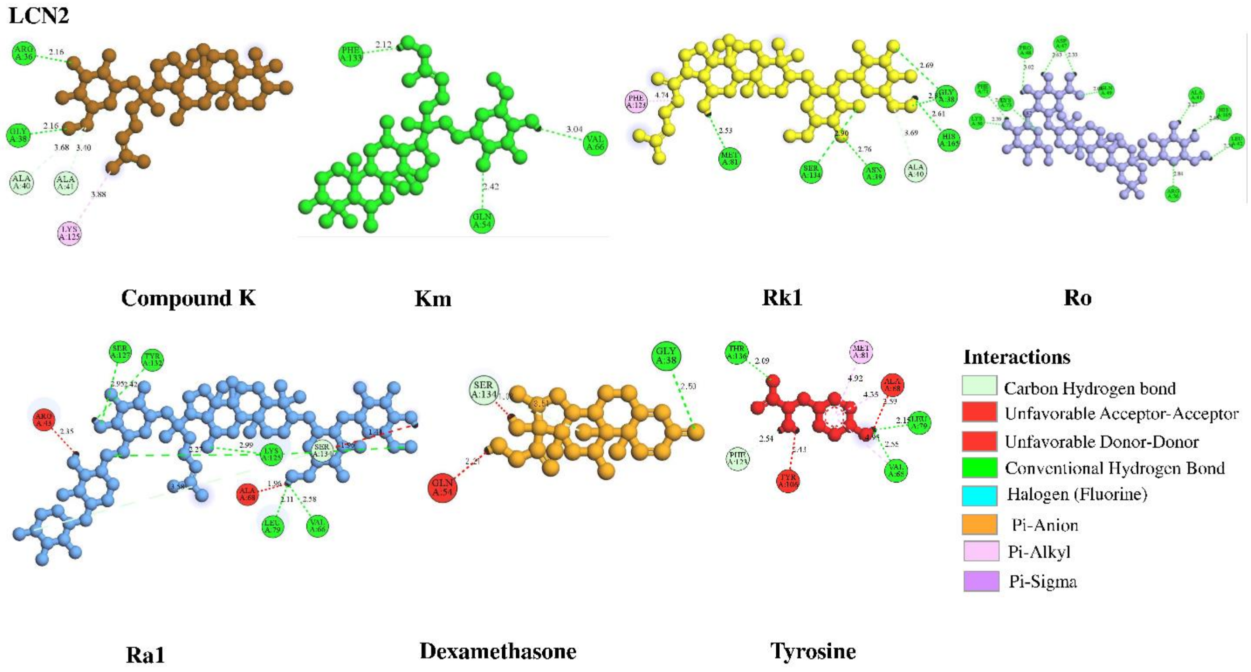

Figure S5. 2D interaction of LCN2 with Compound K, Km, Rk1, Ro, Ra1. Dexamethasone and tyrosine were used as control drugs.

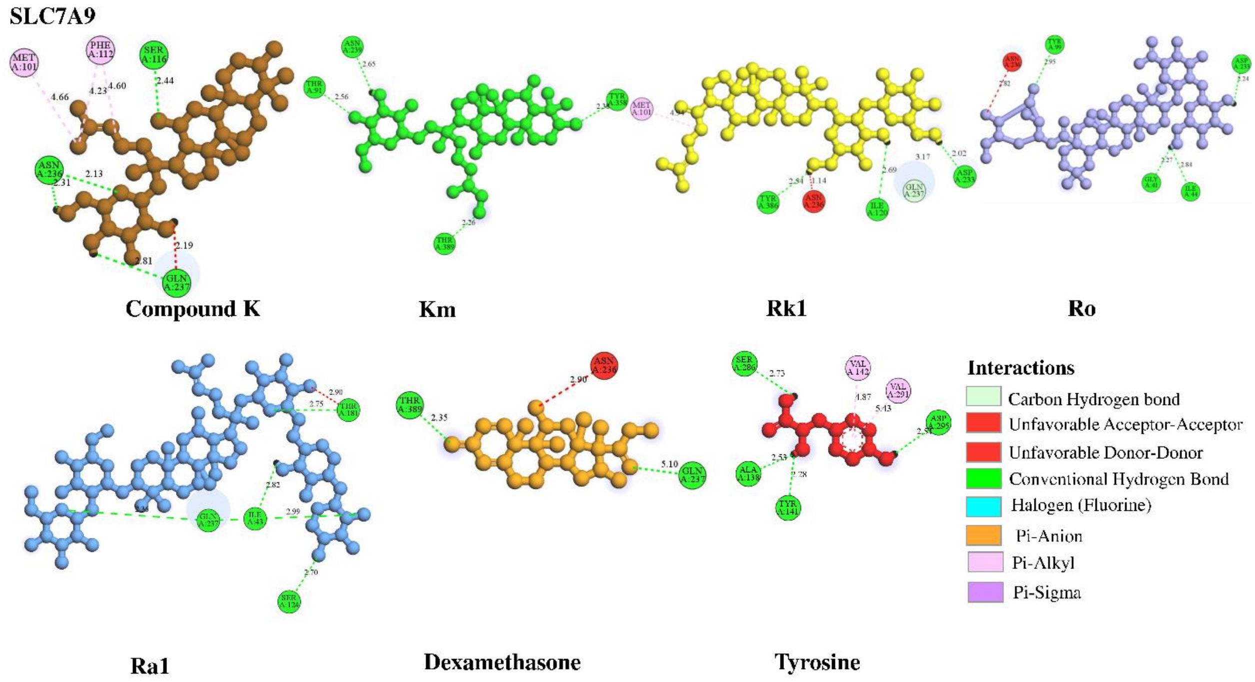

Figure S6. 2D interaction of SLC7A9 with Compound K, Km, Rk1, Ro, Ra1. Dexamethasone and tyrosine were used as control drugs.
